# Glioblastoma Multiforme Cancer Stem Cells Express Components of the Renin–Angiotensin System

**DOI:** 10.3389/fsurg.2016.00051

**Published:** 2016-09-27

**Authors:** Amy Ruth Bradshaw, Agadha Crisantha Wickremesekera, Helen D. Brasch, Alice M. Chibnall, Paul F. Davis, Swee T. Tan, Tinte Itinteang

**Affiliations:** ^1^Gillies McIndoe Research Institute, Wellington, New Zealand; ^2^Department of Neurosurgery, Wellington Regional Hospital, Wellington, New Zealand; ^3^Wellington Regional Plastic, Maxillofacial and Burns Unit, Hutt Hospital, Wellington, New Zealand

**Keywords:** glioblastoma multiforme, cancer, stem cells, renin–angiotensin system

## Abstract

**Aim:**

To investigate the expression of the renin–angiotensin system (RAS) in cancer stem cells (CSCs), we have previously characterized in glioblastoma multiforme (GBM).

**Methods:**

3,3-Diaminobenzidine (DAB) immunohistochemical (IHC) staining for the stem cell marker, SOX2, and components of the RAS: angiotensin converting enzyme (ACE), (pro)renin receptor (PRR), angiotensin II receptor 1 (ATIIR1), and angiotensin II receptor 2 (ATIIR2) on 4 μm-thick formalin-fixed paraffin-embedded sections of previously characterized GBM samples in six patients was undertaken. Immunofluorescent (IF) IHC staining was performed to demonstrate expression of GFAP, SOX2, PRR, ACE, ATIIR1, and ATIIR2. The protein expression and the transcriptional activities of the genes encoding for ACE, PRR, ATIIR1, and ATIIR2 were studied using Western blotting (WB) and NanoString gene expression analysis, respectively.

**Results:**

DAB and IF IHC staining demonstrated the expression SOX2 on the GFAP+ GBM CSCs. Cytoplasmic expression of PRR by the GFAP+ CSCs and the endothelium of the microvessels was observed. ACE was expressed on the endothelium of the microvessels only, while nuclear and cytoplasmic expression of ATIIR1 and ATIIR2 was observed on the endothelium of the microvessels and the CSCs. ATIIR1 was expressed on the GFAP+ CSCs cells, and ATIIR2 was expressed by the SOX2+ CSCs. The expression of ACE, PRR, and ATIIR1, but not ATIIR2, was confirmed by WB. NanoString gene analysis demonstrated transcriptional activation of ACE, PRR, and ATIIR1, but not ATIIR2.

**Conclusion:**

This study demonstrated the expression of PRR, ATIIR1, and ATIIR2 by the SOX2 CSC population, and ACE on the endothelium of the microvessels, within GBM. ACE, PRR, and ATIIR1 were expressed at the protein and mRNA levels, with ATIIR2 detectable only by IHC staining. This novel finding suggests that the CSCs may be a novel therapeutic target for GBM by modulation of the RAS.

## Introduction

Glioblastoma multiforme, a grade IV astrocytoma, contributes to about 50% of all malignant gliomas (1, 2). It almost invariably recurs following surgical resection, radiotherapy, and chemotherapy (3–6). This poor prognosis has been ascribed to the presence of cancer stem cells (CSCs) within GBM, which propagate and differentiate to form downstream cancer cells that make up the bulk of the tumor (7–10).

The CSC concept proposes that a cancer originates from a small population of CSCs, which are generated by upregulation of certain genes in putative resident stem or progenitor cells (11, 12). These genetic alterations confer, upon these cells, the capacity to proliferate and differentiate in an uncontrolled manner resulting in tumorigenesis (11–14). CSCs can be identified using markers associated with embryonic stem cells (ESCs) (15, 16), including ESC markers such as transcription factors NANOG, SALL4, and OCT4, transcription co-factor SOX2 and signaling molecule pSTAT3 (17–21).

Physiologically, the renin–angiotensin system (RAS) is an endocrine system involving conversion of angiotensinogen (ANG) to angiotensin I (ATI) by renin and then to angiotensin II (ATII) by angiotensin converting enzyme (ACE) (22). Renin and its precursor (pro)renin can also bind to the (pro)renin receptor (PRR) to activate MAPK signaling cascades and synthesis of tissue remodeling proteins such as collagen-1, fibronectin, PAI-1, and TGFβ-1 (23–26). Interestingly, the binding of (pro)renin to the PRR also enables conformational activation of the (pro)renin to renin, thereby suggesting the enzyme-like activity of PRR (27).

It has been proposed that ATIIR1 and ATIIR2 are mutually antagonistic in their actions (28–30). There is evidence indicating that ATIIR1 and ATIIR2 play key roles in determining stem cell lineages (31, 32). Inhibition of binding of ATII to either ATIIR1 or ATIIR2 reveals that human hemangioblasts differentiate into either hematopoietic or endothelial progenitor cells depending on whether the signal was transmitted through ATIIR1 or ATIIR2 (31), indicating that the RAS can directly influence stem cell differentiation patterns.

The expression of ANG, (pro)renin, ACE, ATII, ATIIR1, and ATIIR2 has been reported in GBM in humans (33), and components of the RAS may be present on CSCs within this tumor (31, 33, 34).

We have recently demonstrated the presence of CSCs by their expression of the ESC markers NANOG, OCT4, SALL4, pSTAT3, and SOX2 within the GFAP+ GBM tumor samples (35). The aim of this study was to investigate if components of the RAS, namely PRR, ACE, ATIIR1, and ATIIR2 were expressed by this CSC population within GBM.

## Materials and Methods

### Tissue Samples

Six previously characterized GBM tissue samples (35) from 3 male and 3 female patients aged 42–81 years (mean, 64.2 years) were sourced from the Gillies McIndoe Research Institute Tissue Bank, for this study, which was approved by the Central Health and Disabilities Ethics Committee (ref. no. 15CEN28).

### Histology and Immunohistochemical Staining

Four micrometer-thick formalin-fixed paraffin-embedded sections of GBM from six patients were used for hematoxylin and eosin (H&E) staining confirming the presence of GBM by an anatomical pathologist (HDB). Immunohistochemical (IHC) staining was performed on the Leica Bond Rx autostainer (Leica, Nussloch, Germany) as previously described (36). 3,3-Diaminobenzidine (DAB) IHC staining for SOX2 (1:500; cat# PA094, Thermo Fisher, Scientific, Scoresby, VIC, Australia), PRR (1:2000; cat# ab40790, Abcam, Cambridge, UK), ATIIR1 (1:30; cat# ab9391, Abcam), ATIIR2 (1:2000; cat# NBP1-77368, Novus Biologicals, LLC, Littleton, CO, USA), ACE (1:100; cat# MCA2054, AbD Serotec, Kidlington, UK) diluted with Bond™ primary antibody diluent (cat# AR9352, Leica) was done for all tissue samples. Immunofluorescent (IF) IHC staining was performed on two representative GBM tissue samples from the original cohort of patients used for DAB IHC staining, using identical primary antibodies and concentrations. Antibodies used for IF IHC detection of PRR and ATIIR2 combinations were Vecta fluor Excel anti-rabbit 594 (ready-to-use; cat# VEDK-1594, Vector Laboratories, CA, USA) and Alexa Fluor anti-mouse 488 (1:500; cat#A21202, Life Technologies, Carlsbad, CA, USA). Antibodies for IF IHC staining for ACE and ATIIR1 combinations were Vecta fluor Excel anti-mouse (ready-to-use; cat# VEDK2488, Vector Laboratories) and Alexa Fluor anti-rabbit 594 (1:500; cat# A21207, Life Technologies). All IF IHC-stained slides were mounted using Vectashield HardSet antifade mounting medium with DAPI (Vector Laboratories).

Appropriate positive control human tissues for the primary antibodies were placenta for PRR (37), liver for ATIIR1 (38) and ACE (39), kidney for ATIIR2 (38), and skin for SOX2 (35). A secondary and tertiary only negative control was performed on a GBM sample randomly selected from the original cohort of GBM samples used for DAB IHC staining.

### Image Analysis

All DAB IHC stained-slides were visualized with an Olympus BX53 light microscope (Tokyo, Japan) and images were captured with the CellSens 2.0 software (Olympus). IF IHC-stained slides were viewed, and images were captured using an Olympus FV1200 biological confocal laser scanning microscope (Olympus) with images processed using CellSens Dimension 1.11 2D deconvolution algorithm software (Olympus).

### Western Blotting

Five snap-frozen samples of GBM of the original cohort used for DAB IHC staining were washed in 1× PBS and homogenized in RIPA buffer (cat# R0278, Sigma-Aldrich, St Lewis, MA, USA) supplemented with Halt™ Protease and Phosphatase Inhibitor Cocktail (cat# 1861281, Thermo Scientific, Waltham, MA, USA) and dithiothreitol (DTT) (cat# DTT-RO, Sigma-Aldrich, St Lewis, MA, USA). Protein was precipitated using a Calbiochem^®^ ProteoExtract^®^ Protein Precipitation Kit (cat# 539180, EMD Millipore Corp., Billerice, MA, USA) for 1 h at −20°C, washed and re-suspended in 1× Laemmli sample buffer (cat# 161-0737, Bio-Rad, Hercules, CA, USA) with 1% DTT. Equal amounts of protein were heated at 85°C and separated on Bolt™ 4–12% Bis-Tris Plus gels (cat# NW04120BOX, Invitrogen, Carlsbad, CA, USA) *via* electrophoresis. Separated protein was transferred to a nitrocellulose membrane (cat# IB23001, Life Technologies, Carlsbad, CA, USA) and blocked in 1× TBST containing 2% skim milk powder for 90 min at 4°C. Primary antibody probing for each RAS marker was overnight in TBST at 4°C with the following primary antibodies at the given concentrations: PRR (ATP6IP2, 1:500, cat# ab40790, Abcam, Cambridge, UK), ATIIR1 (AT2R1, 1:500; cat# sc-1173, Santa Cruz, CA, USA), ATIIR2 (1:5000; cat# ab92445, Abcam), and ACE (1:200; cat# sc-12184, Santa Cruz). Secondary antibody probing was in 1× TBST for 1 h at 4°C with goat anti-rabbit HRP (1:10,000; cat# A16110, Thermo Fisher) or donkey anti-goat HRP (1:10,000; cat# ab97120; Abcam). ACE tertiary cascade used a rabbit anti-goat Superclonal™ biotin conjugated secondary antibody (1:20,000; cat# A27013, Thermo Fisher) followed by a Pierce™ Streptavidin Poly HRP (1:5000, cat# 21140, Thermo Fisher) at 4°C for 10 min. β-actin antibody probing was performed with the iBind™ Flex device (cat# SLF2000, Life Technologies) using primary mouse monoclonal anti-β-actin (1:2000 cat# ab8226, Abcam) and secondary donkey anti-mouse Alexa fluor 488 (1:2000; cat# A21202, Thermo Fisher). Clarity Western ECL (cat# 1705061, Bio-Rad) was used as the substrate for visualizing HRP detected protein bands, and the Chemi Doc MP Imaging System (Bio-Rad) and Image Lab 5.0 software (Bio-Rad) were used for both HRP and fluorescent band detection and analysis. Appropriate positive controls were human placenta for PRR (37) and ATIIR1 (40), PC3 cell lysate for ATIIR2 (41), and mouse lung for ACE (42). Negative controls were NTERA2 for ATIIR2, HeLa cell lysate for ACE, and no negative tissues or lysates could be found for either the PRR or ATIIR1.

### Nanostring Gene Expression Analysis

Total RNA was extracted from ~20 mg of snap-frozen GBM tissue (*n* = 6) from the same cohort of patients included in DAB IHC staining using the MagJET RNA kit (cat# k2731, Thermo Scientific) and the Kingfisher Duo RNA extraction machine (Thermo Scientific). All samples were quantitated and quality controlled with the NanoDrop 2000 Spectrophotometer (Thermo Scientific) and the Qubit 2.0 Fluorimeter (Thermo Scientific). The samples with A260/A230 ≥ 1.5 and A260/A280 ~ 2 were used for further analyses. The integrity of the RNA was assessed by the New Zealand Genomics Ltd. (Dunedin, New Zealand) using Agilent 2100 BioAnalyzer (Agilent Technologies). The isolated RNA was then subjected to NanoString nCounter™ Gene Expression Assay (NanoString Technologies, Seattle, WA, USA) as completed by New Zealand Genomics Ltd (Dunedin, New Zealand), according to the manufacturer’s protocol. Probes for the genes encoding the PRR (NM_005765.2), ATIIR1 (NM_000685.3), ATIIR2 (NM_000686.3), ACE (NM_000789.2) and the housekeeping gene, and GAPDH (NM_002046.3) were designed and synthesized by NanoString Technologies. Raw data were analyzed with Microsoft Excel using standard settings and were normalized against the housekeeping genes.

## Results

### 3,3-Diaminobenzidine Immunohistochemical Staining

**Figure 1:**
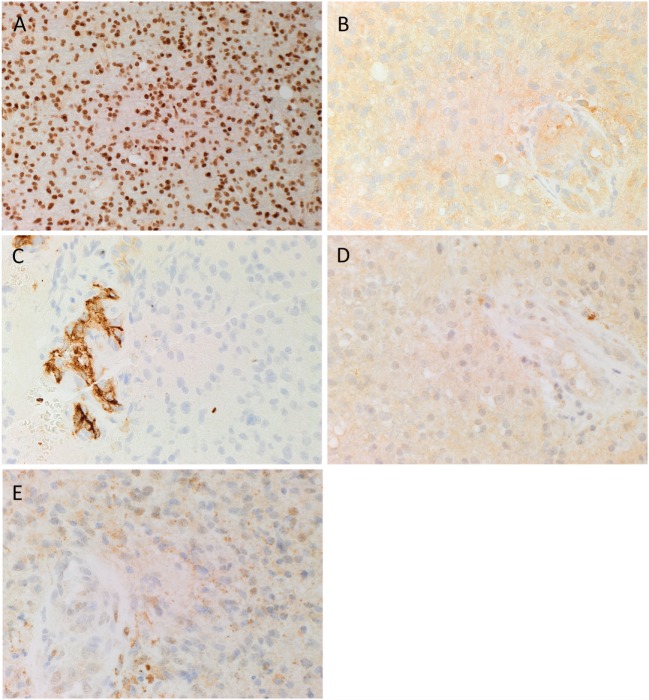
**Representative 3,3-diaminobenzidine immunohistochemical stained images demonstrating cytoplasmic expression of SOX2 [(A), brown], PRR [(B), brown] by cells within GBM, and the endothelium of the microvessels**. ACE [**(C)**, brown] was present only in the endothelium of the microvessels with no staining of the cells within the tumor. Cytoplasmic and nuclear staining of ATIIR1 [**(D)**, brown] and ATIIR2 [**(E)**, brown] was observed on the cells within the tumor and the endothelium of the microvessels. Cell nuclei were counterstained with hematoxylin [**(A–E)**, blue]. Original magnification: 400×.

3,3-Diaminobenzidine IHC staining for SOX2, PRR, ATIIR1, ATIIR2, and ACE was performed on six GBM samples with the diagnosis confirmed by H&E staining. SOX2 was widely expressed by cells within GBM (Figure 1A, brown). These SOX2+ CSCs, that we have previous identified (35), demonstrated cytoplasmic expression of PRR, which was also expressed on the endothelium of the microvessels (Figure 1B, brown). ACE was expressed on the endothelium of the microvessels only, with minimal staining seen on the CSCs (Figure 1C, brown). Nuclear and cytoplasmic expression of ATIIR1 (Figure 1D, brown) and ATIIR2 (Figure 1E, brown) was observed on the endothelium of the microvessels and the CSCs within GBM.

Expected staining patterns for SOX2 (Image S1A in Supplementary Material, brown), PRR (Image S1B in Supplementary Material, brown), ATIIR1 (Image S1C in Supplementary Material, brown), ATIIR2 (Image S1D in Supplementary Material, brown), and ACE (Image S1E in Supplementary Material, brown) were demonstrated in the respective positive controls. Staining with the omission of the primary antibodies in a GBM sample provided an appropriate negative control (Image S1F in Supplementary Material).

### Immunofluorescent Immunohistochemical Staining

**Figure 2:**
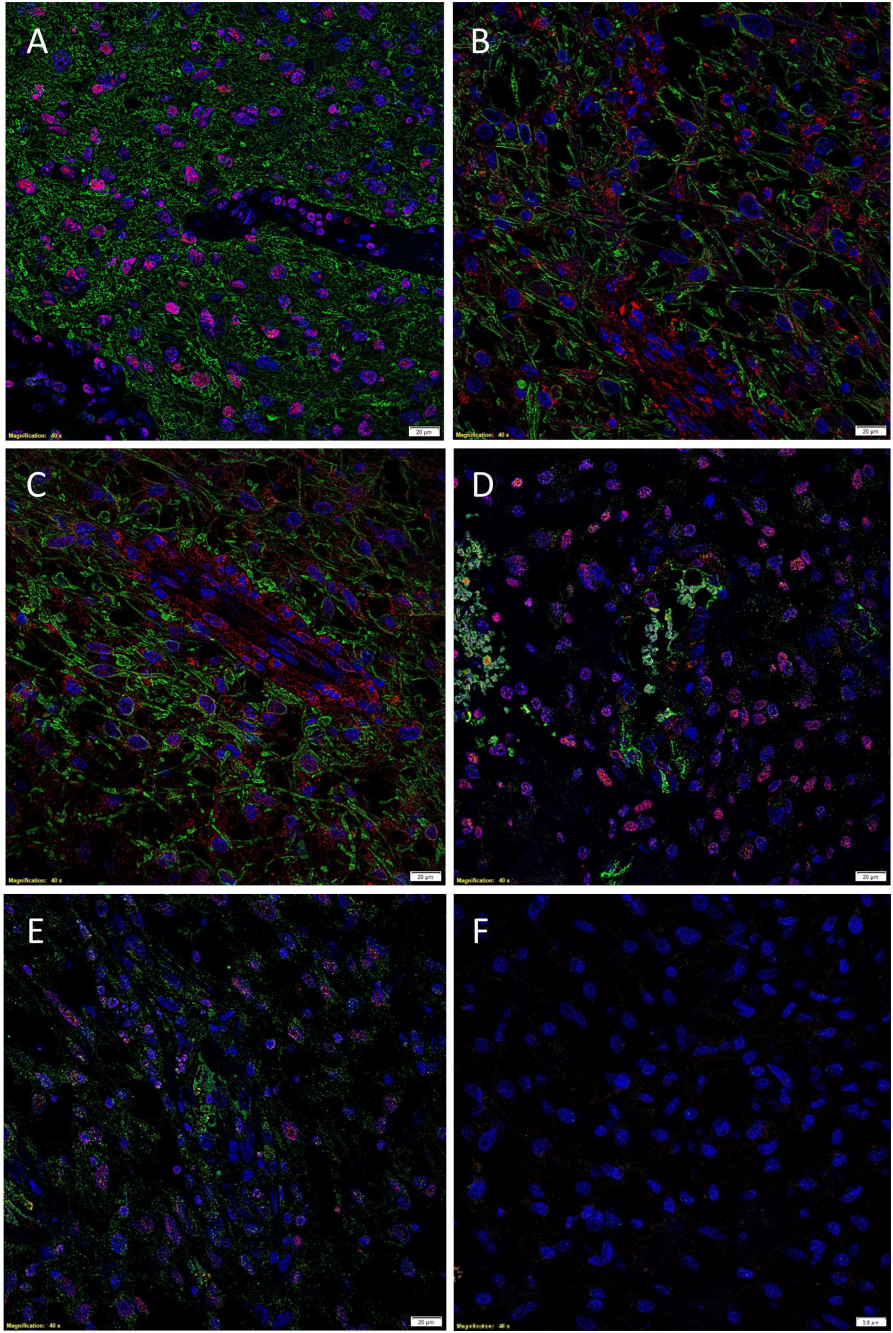
**Representative immunofluorescent immunohistochemical stained images demonstrating the expression of SOX2 [(A), red], PRR [(B), red], and ATIIR2 [(C), red] on GFAP+ CSCs [(A–C), green] and expression of ACE [(D), green] and ATIIR1 [(E), green] on SOX2+ CSCs [(D,E), red]**. Negative control was a GBM tissue section with omission of the primary antibody **(F)**. Cell nuclei were counterstained with 4′, 6′-diamidino-2-phenylindole [**(A–F)**, blue]. Scale bars: 20 μm.

The presence of CSCs within GBM was demonstrated by the relatively abundant expression of the ESC marker SOX2 (Figure 2A, red) on the GFAP+ cells (Figure 2A, green) within GBM, as recently reported (35). We then investigated the expression of PRR (Figure 2B, red) in GBM, by performing IF IHC co-staining with GFAP (Figure 2B, green), which demonstrated that most of the GFAP+ CSCs within GBM expressed PRR. To determine the expression of ACE, we performed dual staining for ACE (Figure 2C, green) and SOX2 (Figure 2C, red) and showed mutually exclusive expression of these markers. Interestingly, ACE was expressed on the endothelial cells with erythrocytes evident within the lumina of the microvessels. We also showed the expression of ATIIR1 (Figure 2D, green) on the SOX2+ (Figure 2D, red) CSC population. ATIIR2 (Figure 2E, red) was expressed on the GFAP+ (Figure 2E, green) CSCs in GBM that were demonstrated to express SOX2 (35). Appropriate negative controls, consisting of omission of the primary antibodies did not reveal any staining (Figure 2F).

### Western Blotting

**Figure 3:**
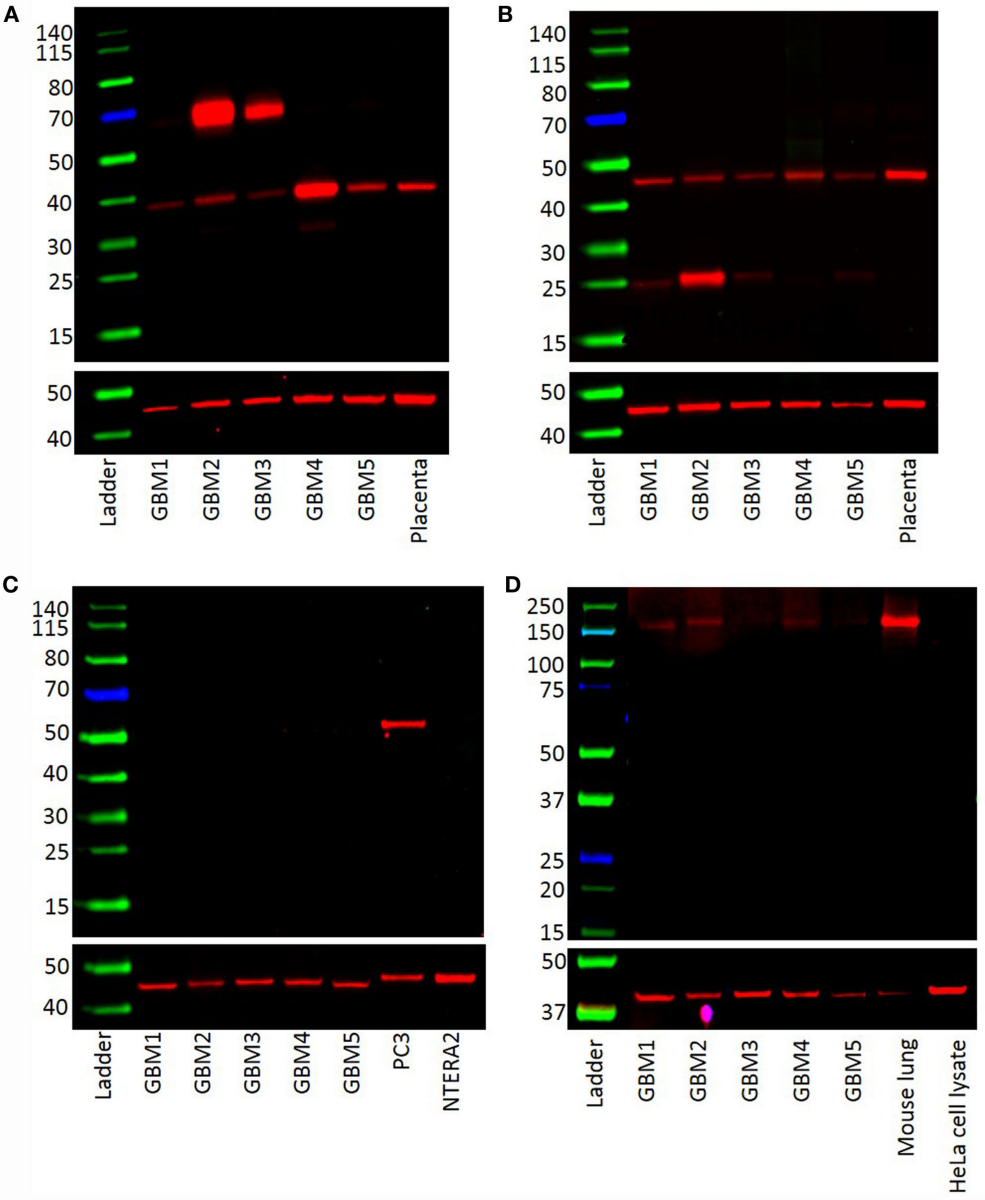
**Western blots demonstrating the expression of PRR (~38 kDa) (A) and ATIIR1 (~45 kDa) (B) in all five GBM samples**. ATIIR2 was not detected in any of the samples **(C)**. ACE was detected in four out of the five GBM samples examined **(D)**.

Western blotting was performed to examine the presence of components of the RAS in GBM samples of five patients included in DAB IHC staining. PRR (Figure 3A) and ATIIR1 (Figure 3B) were present in all five samples with bands of ~37 and 45 kDa, respectively. Bands of ~70 kDa represent PRR dimerization (Figure 3A). ATIIR2 was absent in all five samples (Figure 3C), while ACE was present, at low levels, in all five samples (Figure 3D).

### NanoString Analysis

**Figure 4:**
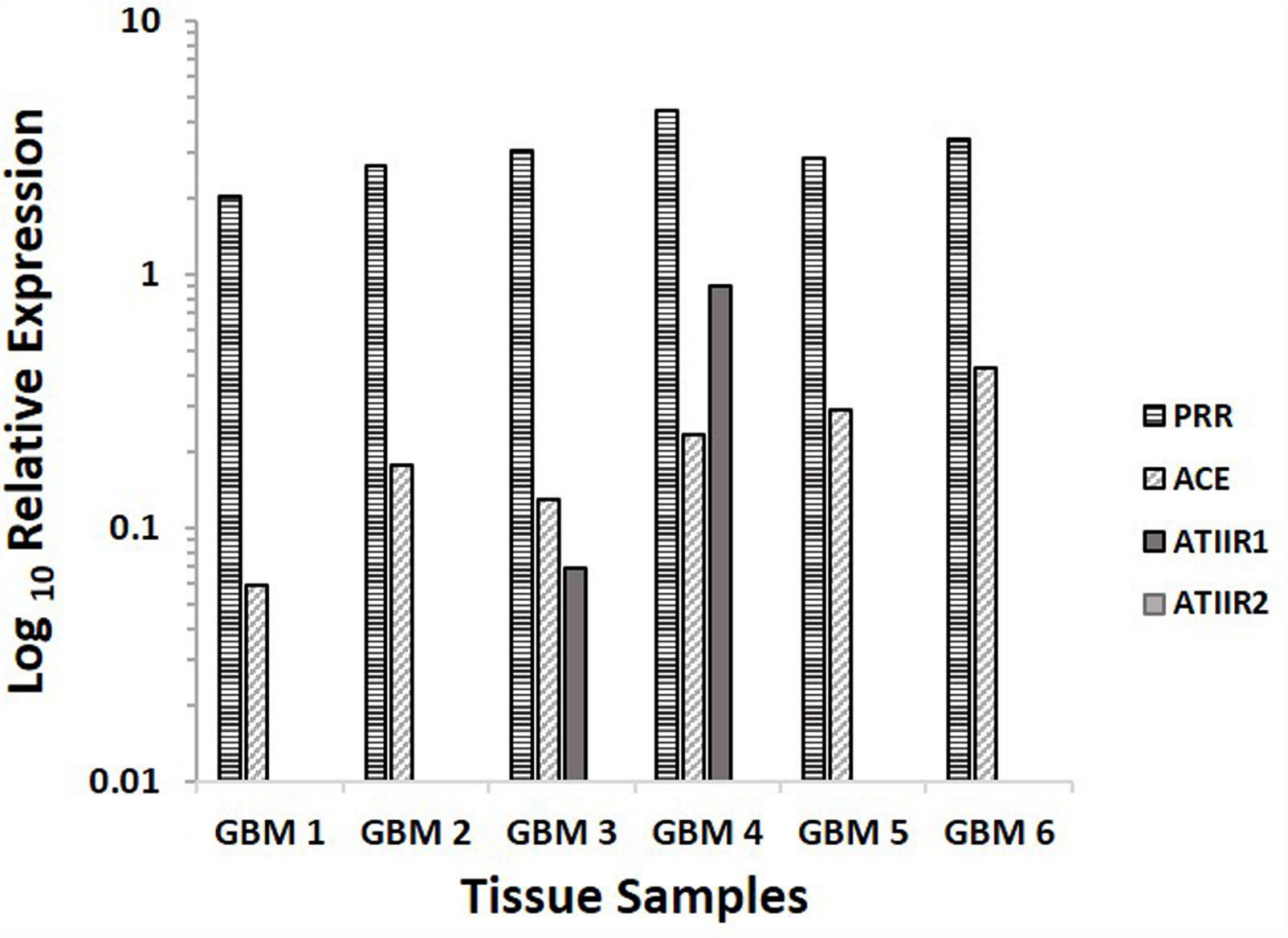
**Relative expression of mRNA transcripts of the components of the RAS in six GBM samples, depicted as a ratio over the GUSB housekeeper**. PRR and ACE were expressed in all six samples. ATIIR1 was present in two and ATIIR2 was below detectable levels out of the six GBM samples examined.

NanoString analyses demonstrated that PRR and ACE were expressed in GBM samples of all six patients included in DAB IHC staining, while ATIIR1 was present in only two samples, and ATIIR2 was below detectable levels in all six samples examined (Figure 4).

## Discussion

Cancer stem cells have been identified in many cancer types (43–48) and were first characterized in GBM by Singh et al. (8, 49). These findings support the CSC concept of cancer proposing that a tumor originates from a small population of cells imbued with the properties of infinite self-renewal and capacity to differentiate into multiple cellular lineages (11–13, 50). Components of the RAS have also been previously identified in GBM (33) and other cancers (34, 51, 52). Additionally, inhibition of the RAS leads to reduced tumor growth indicating a role for the RAS in cancer progression (53–56). We have recently characterized the CSC population within GBM using the ESC markers pSTAT3, SOX2, SALL4, OCT4, and NANOG and demonstrated their relative expression to the GFAP+ cells within GBM tissues (35). Here, we have shown the expression of PRR, ATIIR1, and ACE within GBM tumors at the protein and mRNA levels.

It is intriguing that DAB and IF IHC staining demonstrated the presence of ATIIR2, but this finding was not confirmed by WB and NanoString analyses. This may suggest non-specific binding of the antibody used in DAB and IF IHC staining or, potentially, the splice variants we used did not fully cover ATIIR2. This remains a topic of further investigation.

We have shown that components of the RAS were expressed by the CSCs that we have demonstrated to express SOX2 (35). This finding is particularly interesting when considering the proposed non-angiogenic actions of the RAS. Hemangioblasts are derived from ESCs and are capable of differentiating into either endothelial/vascular or hematopoietic stem cells (57) – an ability directly modulated by differential ATII signaling through either of two receptors, namely ATIIR1 and ATIIR2 (31). The expression of ACE on the endothelium of the microvessels within GBM presented in this report may suggest a putative primitive endothelial phenotype, similar to the expression seen in hemangioblasts (49), and may possibly account for the vascular mimicry previously reported in GBM (58), although this remains the topic of further investigation.

This report demonstrates that components of the RAS are putatively expressed on CSCs within GBM and may dictate cellular commitment to a particular lineage. We and others have proposed a phenotype of the CSCs, similar to ESCs, in GBM (12, 13, 35). This investigation confirms previous reports of expression of components of the RAS in GBM (33). However, based on our recent report of the CSCs in GBM (35), it is noteworthy that the putative CSCs in GBM express certain components of the RAS.

In this report, we show abundant expression of the ESC marker SOX2 on the GFAP+ GBM population, denoting a putative CSC phenotype. Furthermore, we demonstrate the expression of PRR, ATIIR1, and ATIIR2 on most of the GFAP+ CSC population within GBM, with ACE being expressed on the endothelium of the microvessels.

Although this is a relatively small study, the results offer novel insights into the role of the RAS in GBM. It is exciting to speculate that further studies may lead to CSCs in GBM being identified as a potential therapeutic target by modulating the RAS using existing medications.

## Ethics Approval

The study was approved by the Central Health and Disabilities Ethics Committee (ref. no. 15CEN28).

## Author Contributions

TI and ST formulated the study hypothesis. TI, AW, and STT designed the study. TI, HDB, ARB, AW, PFD, and STT interpreted the DAB IHC data. TI, AW, and STT interpreted the IF IHC data. ARB performed WB analysis. ARB, TI, AW, PFD, and STT interpreted the WB data. AMC processed the tissues for NanoString analysis and interpreted the data. ARB, TI, PFD, AW, and STT drafted the manuscript. All authors commented on and approved the manuscript.

## Conflict of Interest Statement

The authors declare that the research was conducted in the absence of any commercial or financial relationships that could be construed as a potential conflict of interest. TI, PFD, and STT are inventors of the PCT patent application (No. PCT/NZ2015/050108) cancer diagnosis and therapy.

## Supplementary Material

Image S1 **DAB IHC stained images of positive control human samples using placenta for SOX2 [(A), brown] and PRR [(B), brown]; liver for ATIIR1 [(C) and brown]; kidney for ATIIR2 [(D), brown] and ACE [(E), brown]**. A GBM tissue section stained in the absence of primary antibody, was used as an appropriate negative control **(F)**. All slides were counterstained with hematoxylin to illustrate cell nuclei (blue). Original magnification: 400×.
