# Supplementary figures and images for: Glioblastoma Multiforme Cancer Stem Cells Express Components of the Renin–Angiotensin System
Source: Front Surg. 2016 Sep 27;3:51. doi: 10.3389/fsurg.2016.00051 (PMC5037176; doi:10.3389/fsurg.2016.00051)

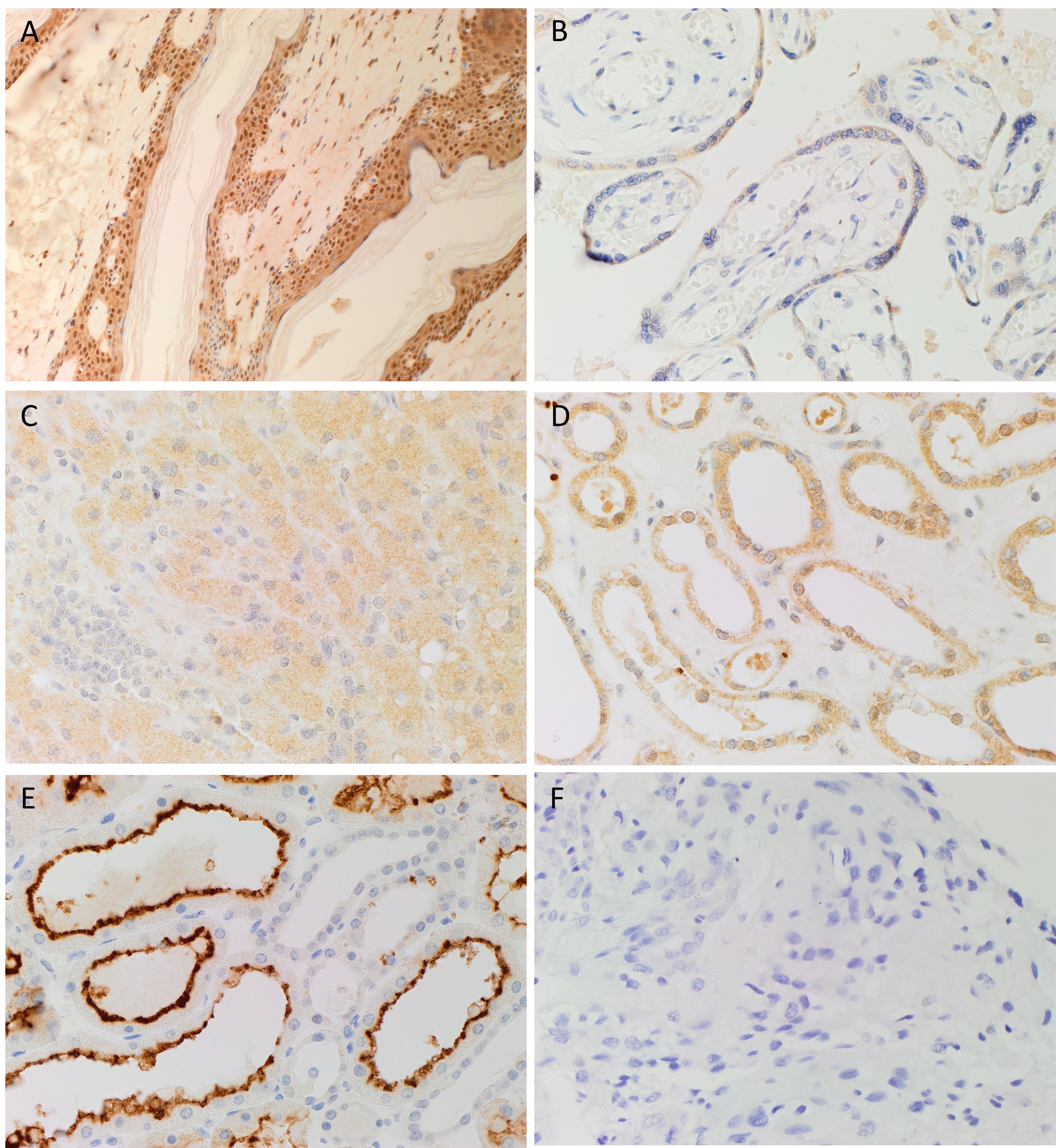

Supplement: Image S1 — DAB IHC stained images of positive control human samples using placenta for SOX2 [(A), brown] and PRR [(B), brown]; liver for ATIIR1 [(C) and brown]; kidney for ATIIR2 [(D), brown] and ACE [(E), brown]. A GBM tissue section stained in the absence of primary antibody, was used as an appropriate negative control (F). All slides were counterstained with hematoxylin to illustrate cell nuclei (blue). Original magnification: 400×. [file Image_1.JPEG]
